# A computationally efficient hybrid 2D–3D subwoofer model

**DOI:** 10.1038/s41598-020-80092-9

**Published:** 2021-01-08

**Authors:** Ahmad H. Bokhari, Martin Berggren, Daniel Noreland, Eddie Wadbro

**Affiliations:** 1 Department of Computing Science, Umeå University, 901 87 Umeå, Sweden; 2 The Forestry Research Institute of Sweden (Skogforsk), Uppsala Science Park, 75183 Uppsala, Sweden

**Keywords:** Applied mathematics, Computational science, Acoustics

## Abstract

A subwoofer generates the lowest frequency range in loudspeaker systems. Subwoofers are used in audio systems for live concerts, movie theatres, home theatres, gaming consoles, cars, etc. During the last decades, numerical simulations have emerged as a cost- and time-efficient complement to traditional experiments in the design process of different products. The aim of this study is to reduce the computational time of simulating the average response for a given subwoofer design. To this end, we propose a hybrid 2D–3D model that reduces the computational time significantly compared to a full 3D model. The hybrid model describes the interaction between different subwoofer components as interacting modules whose acoustic properties can partly be pre-computed. This allows us to efficiently compute the performance of different subwoofer design layouts. The results of the hybrid model are validated against both a lumped element model and a full 3D model over a frequency band of interest. The hybrid model is found to be both accurate and computationally efficient.

## Introduction

The subwoofer is responsible for reproduction of the lowest frequencies in a loudspeaker system. The lower frequency limit for human hearing is around 20 Hz, corresponding to a wavelength of 17 m. This means that the characteristic dimensions of any reasonably sized subwoofer are much smaller than the wavelength. For mathematical modelling, the acoustically small dimensions of a subwoofer justify the use of lumped elements, which are both computationally efficient and conceptually easy to grasp. Lumped elements do have their limitations also for acoustically small components, however. An underlying assumption is that each component (such as the speaker diaphragm, the front chamber, or the ports) of the subwoofer has its own acoustic identity that can be computed separately and that this identity is preserved regardless of the other components. This is not the case if the components are situated so close to each other that their acoustic near-fields interlace. In order to make precise modelling, it is necessary to resort to 3D models of the wave propagation in certain regions. Several commercial software packages that integrate CAD and electroacoustics exist, but while they are convenient for analysing a certain design, computations are time-consuming and therefore impractical if, for example, used in an optimisation loop. Each new layout requires a solution of the governing equation for each frequency of interest. To reduce the computational burden, we propose a hybrid mathematical model that significantly reduces the computational cost of evaluating the subwoofer’s performance. The rationale is to pre-compute the properties of fixed parts of the domain so that these parts can be represented by acoustic boundary, or interface, conditions. Then, only part of the domain subject to change is computed in 2D whereas fixed parts of the domain, such as the port to the exterior and the transducer’s chamber are computed in 3D. The interface conditions are computed for a surface with a resolution sufficiently high to account also for near field effects.

In this study, we use a fourth order bandpass design^1,2^ for the subwoofer. In this design, the transducer is mounted in a sealed back chamber and projects into a ported front chamber, which acts as a passive low pass acoustic filter and only allows a band of frequencies to pass through the output port. The passive acoustic filter has the advantage over an electric filter of being placed so that distortion by the transducer can be filtered out. For more details on bandpass design, readers are referred to Collom’s book^3^ [p 169] on loudspeaker design. In the following sections, we present three subwoofer models in decreasing order of complexity: 3D, hybrid, and lumped. We validate the results of the hybrid model against the full 3D model and also compare them to a classical lumped element model to assess the hybrid model’s efficiency and accuracy.

## 3D mathematical model

Here, we present a full 3D model that describes the interaction between the subwoofer’s different parts. The model uses linear electrical and mechanical circuit models for the transducer, a stiff approximation of the loudspeaker diaphragm, and linear full-wave air acoustics. Such a model is based on fundamental principles of operation for a subwoofer and serves as a stepping stone for all further work. The model thus takes full-wave effects into account but is restricted to the linear working regime, which means that the model will be accurate in the small-signal regime. Of nonlinear effects, those related to air acoustics are by no means the most important in a loudspeaker. Instead, displacement-varying stiffness of the diaphragm, magnetic saturation, and the coil leaving the homogeneous region of the magnetic field are examples of nonlinear effects that are more important when leaving the small-signal regime. Another effect not considered here is the modal breakup of the cone. Note that all these complications concern the transducer only and not the 2D/3D effects of air acoustics, which is the main concern here.

**Figure 1:**
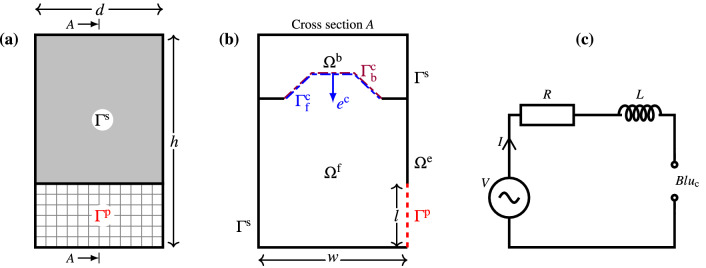
3D mathematical model: (**a**) The subwoofer from the front. (**b**) The cross section of the subwoofer. (**c**) The electric circuit that is part of the electromechanical model of the transducer.

We consider a subwoofer box standing on an infinite floor as illustrated in Fig. 1. The subwoofer box has dimensions $w \times h \times d$, where a transducer is mounted in a sealed back chamber, and an output port of dimensions $l \times d$ is placed in the front chamber. The downward facing transducer is mounted at the centre of a baffle with dimensions $w \times d$.

The back chamber, the front chamber, and the exterior of the subwoofer, as illustrated in Fig. 1b, are denoted by $\Omega ^\text {b}$, $\Omega ^\text {f}$, and $\Omega ^\text {e}$, respectively, and $\Omega ^\text {b}\cup \Omega ^\text {f}$ is denoted by $\Omega$. Let $\Gamma ^\text {p}$ be the boundary that corresponds to the output port, $\Gamma ^\text {s}$ be all wall boundaries, and $\Gamma ^\text {c}$ be the boundary corresponding to the transducer’s cone. Let $e^\text {c}$ be a unit vector in the axial direction of the speaker pointing into $\Omega ^\text {f}$. The front and back side of the speaker cone are denoted $\Gamma _\text {f}^\text {c}$ and $\Gamma _\text {b}^\text {c}$, respectively.

We consider time harmonic wave propagation in the linear regime, where the acoustic pressure is given by $P(x,t)=\mathfrak {R}\left\{ \mathrm {e}^{\mathrm {i}\omega t} p(x)\right\}$, in which $\omega$ denotes the angular frequency, *t* the time, and *p* the complex pressure amplitude. Under these assumptions, *p* satisfies Helmholtz equation in $\Omega \cup \Omega ^e$

1 $$\begin{aligned} k^2p+\Delta p=0, \end{aligned}$$

where *c* is the speed of sound, $k=\omega /c$ is the wave number, and $\Delta =\nabla \cdot \nabla$ is the Laplace operator.

We assume that there is no external source and that the complex amplitude *p* satisfies the Sommerfeld radiation condition, that is

2 $$\begin{aligned} \lim _{\left| x\right| \rightarrow + \infty } \left| x\right| \left( \frac{x}{\left| x\right| } \cdot \nabla p\left( x\right) + \mathrm {i}kp\left( x\right) \right) = 0, \end{aligned}$$

uniformly in all directions.

The acoustic pressure is related to the acoustic velocity field *u* through the linearised Euler equation

3 $$\begin{aligned} \mathrm {i}k \rho c u + \nabla p= 0, \end{aligned}$$

where $\rho$ is the static air density. Evaluating Eq. (3) at a point on the boundary $\partial \Omega$ with outward directed normal *n* yields

4 $$\begin{aligned} \frac{\partial p}{\partial n} = - \mathrm {i} k \rho c n \cdot u. \end{aligned}$$

Expression (4) can be used to assign suitable boundary and interface conditions.

Assuming all walls to be sound-hard, that is, equipped with boundary condition $n\cdot u=0$, Eq. (4) implies the condition

5 $$\begin{aligned} \frac{\partial p}{\partial n} = 0 \quad \hbox { on}\ \Gamma ^\text {s}. \end{aligned}$$

We assume a stiff cone with motion only in the $e^\text {c}$ direction illustrated in Fig. 1b. At the cone boundary, the normal component of the acoustic velocity will agree with the normal component of the cone velocity. Equation (4) then yields conditions

6 $$\begin{aligned} \frac{\partial p^\text {b}}{\partial n^\text {b}} + \mathrm {i}k\rho c u^\text {c} e^\text {c} \cdot n^\text {b} =0 \quad \text {on } \Gamma ^\text {c}_\text {b} \quad \text {and} \quad \frac{\partial p^\text {f}}{\partial n^\text {f}} + \mathrm {i}k\rho c u^\text {c} e^\text {c} \cdot n^\text {f} =0 \quad \text {on } \Gamma ^\text {c}_\text {f}, \end{aligned}$$

where $p^\text {b}$ and $p^\text {f}$ are the complex pressure amplitude in $\Omega ^\text {b}$ and $\Omega ^\text {f}$, respectively, and $n^\text {b}$ and $n^\text {f}=-n^\text {b}$ are outward directed unit normals on $\Gamma ^\text {c}$ with respect to $\Omega ^\text {b}$ and $\Omega ^\text {f}$, respectively. Note that $u^\text {c}$ is the velocity amplitude, and the cone velocity is $u^\text {c}e^\text {c}$.

Next, we consider a lumped mechanical model of the transducer. By Newton’s second law, the displacement amplitude $\delta _c$ of the cone satisfies

7 $$\begin{aligned} \left( -\omega ^2 M_\text {md} + \mathrm {i} \omega R_\text {ms} + \frac{1}{C_\text {ms}}\right) \delta _c = BlI + \int _{\Gamma ^\text {c}} e^\text {c}\cdot (n^\text {b} p^\text {b} + n^\text {f} p^\text {f}), \end{aligned}$$

where $M_{\text {md}}$ is the moving mass of the cone, $R_{\text {ms}}$ is the cone’s mechanical resistance, $C_{\text {ms}}$ is the cone’s mechanical compliance, and *Bl* and *I* denote the force factor and the current, respectively. Multiplying equation (7) by $\mathrm {i}\omega$ and using that $u^\text {c}=\mathrm {i}\omega \delta _\text {c}$, yields

8 $$\begin{aligned} \left( -\omega ^2 M_\text {md} + \mathrm {i} \omega R_\text {ms} + \frac{1}{C_\text {ms}}\right) u^\text {c} = \mathrm {i} \omega \left[ BlI + \int _{\Gamma ^\text {c}} e^\text {c}\cdot (n^\text {b} p^\text {b} + n^\text {f} p^\text {f})\right] . \end{aligned}$$

To close the system, the electromechanical coupling is modelled using the simple electric circuit illustrated in Fig. 1c, assuming that the amplifier delivers a constant voltage *V*. That is,

9 $$\begin{aligned} (R+\mathrm {i}\omega L)I + Bl u^\text {c} = V, \end{aligned}$$

where *R* and *L* denote the resistance and inductance of the voice coil.

### Discretisation

#### Port treatment

Helmholtz equation (1) holds inside the subwoofer as well as in the infinite exterior domain $\Omega ^\text {e}$. However, for computational efficiency, we truncate the domain, compute only in the bounded domain $\Omega$, and represent the response from the exterior through a boundary condition on $\Gamma ^\text {p}$. For this, we split the boundary $\Gamma ^\text {p}$ into $N^\text {p}=N^\text {p}_h\times N^\text {p}_d$ square panels, $\Gamma ^\text {p}_j$, $j=1,\ldots ,N^\text {p}$ as shown in Fig. 1b and assume that the acoustic velocity is constant on each panel. Physically this assumption corresponds to a port divided into $N^\text {p}$ stiff and mass-less pistons. The exterior response is represented through a square impedance matrix ${\varvec{Z}}^\text {p}_\text {3D}$ of order $N^\text {p}$ that maps the piece-wise constant acoustic velocities to the mean acoustic pressure on each panel. That is,

10 $$\begin{aligned} {\varvec{Z}}_\text {3D}^\text {p} {\varvec{u}}^\text {p} = {\varvec{p}}^\text {p}, \end{aligned}$$

where ${\varvec{u}}^\text {p}= \big [u^\text {p}_1, u^\text {p}_2,\ldots , u^\text {p}_{N^\text {p}}\big ]^T$ and ${\varvec{p}}^\text {p} = \big [\langle p\rangle _{\Gamma ^\text {p}_1},\langle p\rangle _{\Gamma ^\text {p}_2}, \ldots , \langle p\rangle _{\Gamma ^\text {p}_{N^\text {p}}} \big ]^T$, where $u_j^\text {p}$ is the velocity amplitude in direction $n^\text {p}$ on $\Gamma ^\text {p}_j$, and $\langle p\rangle _{\Gamma ^\text {p}_j}$ is the average pressure on $\Gamma ^\text {p}_j$. For the numerical experiments in this paper, we use piecewise constant boundary elements to approximate the problem. More precisely, to assemble the impedance matrix ${\varvec{Z}}_\text {3D}^\text {p}$, we solve $N^\text {p}$ exterior problems by the boundary element method^4^. The algorithm for calculating the impedance matrix, column by column, is as follows:

For $$j=1,2,\ldots , N^\text {p}$$

- Set ${\varvec{u}}^\text {p}$ to the $j\text {th}$ unit vector, this corresponds to setting $n \cdot u = 1$ on $\Gamma _j^\text {p}$ and $n \cdot u = 0$ on $\partial \Omega \backslash \Gamma _j$.
- Solve Helmholtz equation (1) in $\Omega ^\text {e}$ with boundary condition (4) on $\partial \Omega$ and the Sommerfeld radiation condition (2).
- Evaluate the average pressure on $\Gamma ^\text {p}_i$, for $i=1,\ldots ,N^\text {p}$; the vector of average pressures $\big [\langle p\rangle _{\Gamma ^\text {p}_1},\langle p\rangle _{\Gamma ^\text {p}_2}, \ldots , \langle p\rangle _{\Gamma ^\text {p}_{N^\text {p}}} \big ]^T$ is the $j\text {th}$ column of the matrix ${\varvec{Z}}_\text {3D}^\text {p}$.

#### Finite element discretisation of the subwoofer’s interior

Multiplying Helmholtz equation (1) by test functions $q^\text {f}$ and $q^\text {b}$, integrating over $\Omega ^\text {f}$ and $\Omega ^\text {b}$, respectively, applying integration by parts, and using the boundary conditions (4), (5), and (6), we obtain

11 $$\begin{aligned} \int _{\Omega ^\text {f}} \nabla q^\text {f} \cdot \nabla p^\text {f} -k^2 \int _{\Omega ^\text {f}} q^\text {f} p^\text {f} - \mathrm {i} k \int _{\Gamma ^\text {p}} \rho c u^\text {p}\cdot n^\text {p} q^\text {f} - \mathrm {i} k \int _{\Gamma ^\text {c}_\text {f}}\rho c u^\text {c} e^\text {c}\cdot n^\text {f} q^\text {f} = 0, \quad \forall q^\text {f}\in H^1(\Omega ^\text {f}), \end{aligned}$$

and

12 $$\begin{aligned} \int _{\Omega ^\text {b}} \nabla q^\text {b} \cdot \nabla p^\text {b} -k^2 \int _{\Omega ^\text {b}} q^\text {b} p^\text {b} - \mathrm {i} k \int _{\Gamma ^\text {c}_\text {b}}\rho c u^\text {c} e^\text {c} \cdot n^\text {b} q^\text {b} = 0, \quad \forall q^\text {b}\in H^1(\Omega ^\text {b}). \end{aligned}$$

We will apply the finite element method (FEM) to solve discretised versions of the problems (11) and (12) in domains $\Omega ^\text {f}$ and $\Omega ^\text {b}$, respectively. Note that problems (11) and (12) are coupled through the cone velocity on $\Gamma ^\text {c}$.

We triangulate $\Omega ^\text {f}$ and $\Omega ^\text {b}$, respectively, and remark that the meshes on $\Omega ^\text {f}$ and $\Omega ^\text {b}$ do not need to match on their common interface. For the numerical experiments in this paper, we use second order Lagrange basis functions, also known as 10-node tetrahedron elements. We approximate the complex amplitude function of pressure $p^\text {f}$ and $p^\text {b}$, and the test functions $q^\text {f}$ and $q^\text {b}$ by

13 $$\begin{aligned} \quad p^\text {f}_h = \sum _{j=1}^{N^\text {f}} p^\text {f}_j \theta _j, \quad q^\text {f}_h=\sum ^{N^\text {f}}_{i=1} q^\text {f}_i \theta _i, \quad p^\text {b}_h = \sum _{j=1}^{N^\text {b}} p^\text {b}_j \varphi _j, \quad \text {and} \quad q^\text {b}_h=\sum ^{N^\text {b}}_{i=1} q^\text {b}_i \varphi _i. \end{aligned}$$

where $N^\text {f}$ and $N^\text {b}$ are the number of degrees of freedom for the finite element approximation in $\Omega ^\text {f}$ and $\Omega ^\text {b}$, respectively. In the expressions above $\theta _1,\theta _2,\ldots ,\theta _{N^\text {f}}$ denote the basis functions in $\Omega ^\text {f}$, and $\varphi _1,\varphi _2,\ldots ,\varphi _{N^\text {b}}$ denote the basis functions in $\Omega ^\text {b}$.

To describe the subwoofer system, we need to determine ${\varvec{x}}=\big [\left( {\varvec{p}}^\text {f}\right) ^T,\,\left( {\varvec{p}}^\text {b}\right) ^T,\,\left( {\varvec{u}}^\text {p}\right) ^T,\,u^\text {c},\,I \big ]^T$, where ${\varvec{p}}^\text {f} = \left[ p_1^\text {f}, p_2^\text {f}, \ldots , p^\text {f}_{N^\text {f}}\right] ^T$, ${\varvec{p}}^\text {b} = \left[ p_1^\text {b}, p_2^\text {b}, \ldots , p^\text {b}_{N^\text {b}}\right] ^T$, and ${\varvec{u}}^\text {p}$, $u^\text {c}$, and *I* are defined as above. The discretised form of Eqs. (11) and (12) are

14 $$\begin{aligned} \sum ^{N^\text {f}}_{j=1}\left( \int _{\Omega ^\text {f}}\nabla \theta _i \cdot \nabla \theta _j- k^2\int _{\Omega ^\text {f}} \theta _i \theta _j\right) p^\text {f}_j - \mathrm {i} k \rho c \sum ^{N^\text {p}}_{j=1} u_{j}^\text {p} \int _{\Gamma _j^\text {p}} \theta _i -\mathrm {i} k \rho c u^\text {c} \int _{\Gamma ^\text {c}} e^\text {c} \cdot n^\text {f} \theta _i=0, \quad i=1,\, 2,\, \ldots ,\, N^\text {f}, \end{aligned}$$

and

15 $$\begin{aligned} \sum ^{N^\text {b}}_{j=1}\left( \int _{\Omega ^\text {b}}\nabla \varphi _i \cdot \nabla \varphi _j-k^2\int _{\Omega ^\text {b}} \varphi _i \varphi _j\right) p^\text {b}_j -\mathrm {i}k \rho c u^\text {c} \int _{\Gamma ^\text {c}} e^\text {c}\cdot n^\text {b} \varphi _i=0, \quad i=1,\,2,\,\ldots ,\,N^\text {b}. \end{aligned}$$

Equation (14) is equivalent to

16 $$\begin{aligned} \begin{bmatrix} {\varvec{A}}^\text {ff}&{\varvec{0}}_{N^\text {f}\times N^\text {b}}&{\varvec{A}}^\text {fp}&{\varvec{a}}^\text {fc}&{\varvec{0}}_{N^\text {f}\times 1} \end{bmatrix} {\varvec{x}} = {\varvec{0}}_{N^\text {f}\times 1}, \end{aligned}$$

where the $N^\text {f}\times N^\text {f}$ matrix ${\varvec{A}}^\text {ff}$, the $N^\text {f}\times N^\text {p}$ matrix ${\varvec{A}}^\text {fp}$, and the $N^\text {f}\times 1$ vector ${\varvec{a}}^\text {fc}$ have entries

17 $$\begin{aligned} a^\text {ff}_{ij} = \int _{\Omega ^\text {f}} \nabla \theta _i \cdot \nabla \theta _j -k^2\int _{\Omega ^\text {f}} \theta _i \theta _j, \quad a^\text {fp}_{ij} = -\mathrm {i} k \rho c \int _{\Gamma _j^\text {p}} \theta _i, \quad \text {and} \quad a^\text {fc}_{i} =-\mathrm {i}k \rho c \int _{\Gamma ^\text {c}} e^\text {c}\cdot n^\text {f} \theta _i. \end{aligned}$$

Equation (15) can be written as

18 $$\begin{aligned} \begin{bmatrix} {\varvec{0}}_{N^\text {b}\times N^\text {f}}&{\varvec{A}}^\text {bb}&{\varvec{0}}_{N^\text {b}\times N^\text {p}}&{\varvec{a}}^\text {bc}&{\varvec{0}}_{N^\text {b} \times 1} \end{bmatrix} {\varvec{x}} ={\varvec{0}}_{N^\text {b} \times 1}, \end{aligned}$$

where the $N^\text {b}\times N^\text {b}$ matrix ${\varvec{A}}^\text {bb}$ and the $N^\text {b}\times 1$ vector ${\varvec{a}}^\text {bc}$ have entries

19 $$\begin{aligned} a^\text {bb}_{ij} = \int _{\Omega ^\text {b}} \nabla \varphi _i \cdot \nabla \varphi _j - k^2\int _{\Omega ^\text {b}} \varphi _i \varphi _j \quad \text {and} \quad a^\text {bc}_{i} =-\mathrm {i} k \rho c \int _{\Gamma ^\text {c}} e^\text {c}\cdot n^\text {b} \varphi _i. \end{aligned}$$

To couple $p^\text {f}$ and $u^\text {p}$, we use Eq. (10), which can be written

20 $$\begin{aligned} \begin{bmatrix} {\varvec{A}}^\text {pf}&{\varvec{0}}_{N^\text {p}\times N^\text {b}}&{\varvec{Z}}^\text {p}_\text {3D}&{\varvec{0}}_{N^\text {p}\times 1}&{\varvec{0}}_{N^\text {p}\times 1} \end{bmatrix} {\varvec{x}} = {\varvec{0}}_{N^\text {p}\times 1}, \end{aligned}$$

where the $N^\text {p}\times N^\text {f}$ matrix ${\varvec{A}}^\text {pf}$ has entries

21 $$\begin{aligned} a^\text {pf}_{ij}=\mathrm {i} k \int _{\Gamma ^\text {p}_i}\theta _j. \end{aligned}$$

The discretised form of mechanical equation (8) which models the speaker cone displacement can be written

22 $$\begin{aligned} \begin{bmatrix} {\varvec{a}}^\text {cf}&{\varvec{a}}^\text {cb}&{\varvec{0}}_{1\times N^\text {p}}&-\omega ^2 M_\text {md}+\mathrm {i}\omega R_\text {ms} + \frac{1}{C_\text {ms}}&\mathrm {i}\omega Bl \end{bmatrix} {\varvec{x}} = 0, \end{aligned}$$

where the $1\times N^\text {f}$ vector ${\varvec{a}}^\text {cf}$, and the $1\times N^\text {b}$ vector ${\varvec{a}}^\text {cb}$ have entries

23 $$\begin{aligned} a^\text {cf}_j = -\mathrm {i}\omega \int _{\Gamma ^\text {c}} e^\text {c} \cdot n^\text {f} \theta _j, \quad \text {and} \quad a^\text {cb}_j = -\mathrm {i}\omega \int _{\Gamma ^\text {c}} e^\text {c} \cdot n^\text {b} \varphi _j. \end{aligned}$$

To close the system, we need the electric circuit equation (9). Altogether, the full system in matrix form is

24 $$\begin{aligned} \underbrace{ \begin{bmatrix} {\varvec{A}}^\text {ff} &{} {\varvec{0}} &{} {\varvec{A}}^\text {fp} &{} {\varvec{a}}^\text {fc} &{} {\varvec{0}} \\ {\varvec{0}} &{} {\varvec{A}}^\text {bb} &{} {\varvec{0}} &{} {\varvec{a}}^\text {bc} &{} {\varvec{0}} \\ {\varvec{A}}^\text {pf} &{} {\varvec{0}} &{} {\varvec{Z}}^\text {p}_\text {3D} &{} {\varvec{0}} &{} {\varvec{0}}\\ {\varvec{a}}^\text {cf} &{} {\varvec{a}}^\text {cb} &{} {\varvec{0}} &{} a^\text {cc} &{} a^\text {cI} \\ {\varvec{0}} &{} {\varvec{0}} &{} {\varvec{0}} &{} a^\text {Ic} &{} a^\text {II} \\ \end{bmatrix}}_{{\varvec{A}}} \underbrace{ \begin{bmatrix} {\varvec{p}}^\text {f}\\ {\varvec{p}}^\text {b}\\ {\varvec{u}}^\text {p} \\ u^\text {c}\\ I \end{bmatrix}}_{{\varvec{x}}} = \underbrace{ \begin{bmatrix} {\varvec{0}}\\ {\varvec{0}}\\ {\varvec{0}}\\ 0\\ V \end{bmatrix}}_{{\varvec{b}}}, \end{aligned}$$

where

25 $$\begin{aligned} a^\text {cc}=-\omega ^2 M_\text {md}+\mathrm {i}\omega R_\text {ms} + \frac{1}{C_\text {ms}}, \quad a^\text {cI}= -\mathrm {i}\omega Bl, \quad a^\text {Ic} = \frac{Bl}{\rho c}, \quad \text {and}\quad a^\text {II}= R+\mathrm {i}\omega L. \end{aligned}$$

## Hybrid mathematical model

**Figure 2:**
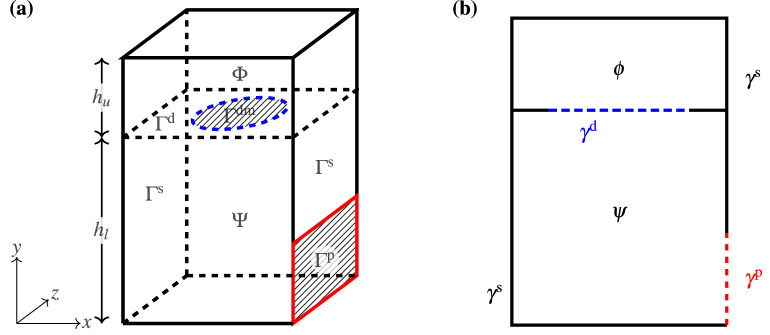
Hybrid mathematical model: (**a**) The subwoofer is divided into two domains, $\Phi$ and $\Psi$. (**b**) Computational domain for the 2D wave propagation within the lower box.

In the hybrid model, we split the interior of the subwoofer into two domains (upper and lower) by a horizontal plane at the baffle. Let $\Gamma ^\text {d}$ correspond to the boundary between these two domains. Figure 2a illustrates that the upper box, whose air region is denoted by $\Phi$, is the region above the baffle and contains the transducer, and that the lower box, denoted by $\Psi$, contains the internal air region below the baffle. Note that any internal walls inside the lower box are not included in $\Psi$; also, we assume that any such walls are fully extruded in the z-direction. In other words, the material layout is planar symmetric along the z-axis. However, in the upper box, the material layout will not be planar-symmetric.

To save computational time, we model the wave propagation within the lower box in 2D. The use of the 2D model for the lower box is justified due to planar symmetry, because the frequencies of interest correspond to wavelengths that are long compared to the lower box, and because the waves sufficiently far away from the cone do not exhibit 3D characteristics. However, we rely on 3D models for the upper box and the exterior since 3D effects cannot be avoided there. The upper box, the lower box, and the exterior are treated as separate modules that interact with each other only through interface conditions. Acoustic properties of the 3D models are pre-computed in the form of impedance matrices, ${\varvec{Z}}^\text {d}$ and ${\varvec{Z}}_\text {2D}^\text {p}$ for the upper box and the exterior, respectively. After we have computed the impedance matrices, the lower box is our computational domain where we solve the system of equations using 2D FEM. Computations for various 2D layouts of material in the lower box will then be very fast.

### Upper box

**Figure 3:**
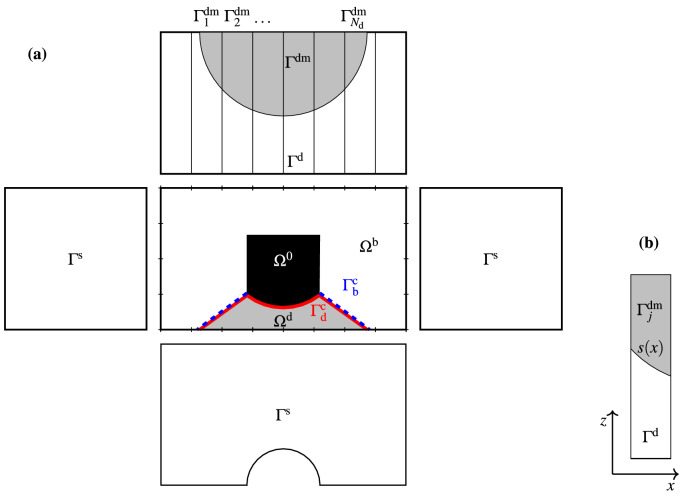
Computational domain for the upper box: (**a**) First-angle projection of the computational domain with the exterior stripes used to compute the impedance matrix ${\varvec{Z}}^\text {d}$. (**b**) Definitions for the computations of boundary integrals associated with the speaker mouth.

Consider an upper box of dimensions $w\times h_\text {u} \times d$. The upper box contains the downward-facing transducer, which is mounted at the centre of a baffle with dimensions $w \times d$. By symmetry, we only compute on half of the upper box of dimension $w \times h_\text {u} \times d/2$. The upper box can be split into three parts, as illustrated in Fig. 3a. The part in front of the speaker cone is denoted $\Omega ^\text {d}$, and the two parts behind the speaker cone are denoted $\Omega ^\text {0}$ and $\Omega ^\text {b}$ respectively. The domain $\Omega ^\text {d}$ contains some part of the domain $\Omega ^\text {f}$ that is above the baffle and in front of the speaker, that is, $\Omega ^\text {d}=\Omega ^\text {f}\backslash \Psi$. The computational domain for the upper box is $\Omega ^\text {b}\cup \Omega ^\text {d}$, and the third region, $\Omega ^0$, is filled with sound-hard material that contains non-moving parts of the transducer. Recall that all walls of the subwoofer are sound-hard; therefore the sides of the upper box facing up, left, right, front and back are included in $\Gamma ^\text {s}$. The side of the upper box facing downward is denoted $\Gamma ^\text {d}$ and it also contains the mouth of the speaker $\Gamma ^\text {dm}\subset \Gamma ^\text {d}$. The grey half-circle in Fig. 3a illustrates the mouth of the speaker $\Gamma ^\text {dm}$. The speaker cone surfaces are denoted $\Gamma ^\text {c} = \Gamma _\text {f}^\text {c} \cup \Gamma _\text {b}^\text {c}$.

The governing equations of the upper box system are the mechanical equation (8), modelling the cone displacement, Helmholtz equation (1) in $\Phi$, conditions for coupling (4) to the lower box at $\Gamma ^\text {dm}$, to the back $\Gamma _\text {b}^\text {c}$ and the front $\Gamma _\text {f}^\text {c}$ of the cone (6), and the sound-hard (5) boundary condition at $\Gamma ^\text {s}$.

The variational forms of the governing equation in $\Omega ^\text {d}\cup \Omega ^\text {b}$, derived the same way as Eqs. (11) and (12), are

26 $$ \int _{\Omega ^\text {d}} \nabla q^\text {d} \cdot \nabla p^\text {d} -k^2\int _{\Omega ^\text {d}} q^\text {d} p^\text {d} - \mathrm {i} k \int _{\Gamma ^\text {dm}} \rho c u^\text {d}\cdot n^\text {d} q^\text {d} - \mathrm {i} k \int _{\Gamma ^\text {c}_\text {f}} \rho c u^\text {c} e^\text {c} \cdot n^\text {f} q^\text {d} = 0, \quad \forall q^\text {d} \in H^1(\Omega ^\text {d}),  $$

and

27 $$ \int _{\Omega ^\text {b}} \nabla q^\text {b} \cdot \nabla p^\text {b} -k^2\int _{\Omega ^\text {b}} q^\text {b} p^\text {b} - \mathrm {i} k \int _{\Gamma ^\text {c}_\text {b}} \rho c u^\text {c} e^\text {c} \cdot n^\text {b} q^\text {b} = 0, \quad \forall q^\text {b} \in H^1(\Omega ^\text {b}).  $$

We triangulate $\Omega ^\text {d}$ and $\Omega ^\text {b}$, respectively, and define $M^\text {d}$ basis functions $\eta _1,\eta _2,\ldots ,\eta _{M^\text {d}}$ in $\Omega ^\text {d}$ and $M^\text {b}$ basis functions $\xi _1,\xi _2,\ldots ,\xi _{M^\text {b}}$ in $\Omega ^\text {b}$. For the numerical experiments in this paper, we use second order Lagrange basis functions. The discretised form of Eqs. (26) and (27) are

28 $$\begin{aligned}&\sum ^{M^\text {d}}_{j=1}\left( \int _{\Omega ^\text {d}}\nabla \eta _i \cdot \nabla \eta _j- k^2\int _{\Omega ^\text {d}} \eta _i \eta _j\right) p^\text {d}_j - \mathrm {i} k \rho c \sum ^{N^\text {d}}_{j=1} u_{j}^\text {d} \int _{\Gamma _j^\text {dm}} \eta _i \nonumber \\&\qquad - \mathrm {i} k \rho c u^\text {c}\int _{\Gamma ^\text {c}_\text {f}} e^\text {c} \cdot n^\text {d} \eta _i=0, \quad i=1,\,2,\,\ldots ,\,M^\text {d}, \end{aligned}$$

and

29 $$\begin{aligned} \sum ^{M^\text {b}}_{j=1}\left( \int _{\Omega ^\text {b}}\nabla \xi _i \cdot \nabla \xi _j- k^2\int _{\Omega ^\text {b}} \xi _i \xi _j\right) p^\text {b}_j -\mathrm {i} k \rho c u^\text {c}\int _{\Gamma ^\text {c}_\text {b}} e^\text {c}\cdot n^\text {b}\xi _i = 0, \quad i=1,\,2,\,\ldots ,\, M^\text {b}. \end{aligned}$$

where $u^\text {d}_j$ is the velocity amplitude in the direction of $n^\text {d}$ on $\Gamma ^\text {dm}_j$.

The mechanical equation (8) for the cone dynamics in its discretised form is

30 $$\begin{aligned} - \mathrm {i}\omega \sum ^{M^\text {d}}_{j=1} p^\text {d}_{j} \int _{\Gamma ^\text {c}_\text {f}}n^\text {d} \cdot e^\text {c} \eta _j- \mathrm {i}\omega \sum ^{M^\text {b}}_{j=1} p^\text {b}_j\int _{\Gamma ^\text {c}_\text {b}} n^\text {b} \cdot e^\text {c} \xi _j+ \left( -\omega ^2 M_\text {md} + i \omega R_\text {ms} + \frac{1}{C_\text {ms}}\right) u^\text {c} - \mathrm {i} \omega BlI =0. \end{aligned}$$

The coupling between *p* and *u* on the boundary $\Gamma ^\text {dm}$ depends on the transducer dynamics that is located inside the upper box. Therefore, the impedance matrix also takes into account the cone velocity and current *I*. The boundary $\Gamma ^\text {dm}$ is divided into $N^\text {d}$ depth running strips illustrated in Fig. 3a. Similarly, as for the port, we assume constant acoustic velocity on each strip, which physically corresponds to a boundary $\Gamma ^\text {dm}$ made up of stiff mass-less pistons. The effects of the upper box are then taken into account by an acoustic impedance relation

31 $$\begin{aligned} \underbrace{ \begin{bmatrix} {\varvec{Z}}^\text {dd} &{} {\varvec{Z}}^\text {cd} \\ {\varvec{Z}}^\text {dc} &{} Z^\text {cc} \end{bmatrix}}_{{\varvec{Z}}^\text {d}} \begin{bmatrix} {\varvec{u}}^\text {d}\\ u^\text {c} \end{bmatrix} = \begin{bmatrix} {\varvec{p}}^\text {d} \\ I \end{bmatrix}, \end{aligned}$$

where ${\varvec{Z}}^\text {dd}$ is $N^\text {d}\times N^\text {d}$, ${\varvec{Z}}^\text {dc}$ is $N^\text {d}\times 1$, ${\varvec{Z}}^\text {cd}$ is $1\times N^\text {d}$, $Z^\text {cc}$ is $1\times 1$, ${\varvec{u}}^\text {d}=\big [u^\text {d}_1,\,u^\text {d}_2,\,\ldots ,\,u^\text {d}_{N^\text {d}}\big ]^T$, and ${\varvec{p}}^\text {d} = \big [\langle p\rangle _{\Gamma ^\text {dm}_1},\langle p\rangle _{\Gamma ^\text {dm}_2}, \ldots , \langle p \rangle _{\Gamma ^\text {dm}_{N^\text {d}}} \big ]^T$, where $\langle p\rangle _{\Gamma ^\text {dm}_j}$ is the average pressure on $\Gamma ^\text {dm}_j$. The matrix ${\varvec{Z}}^\text {d}$ maps the $N^\text {d}+1$ vector of panel velocities and cone velocity to the $N^\text {d}+1$ vector of average pressures on the panels and the voice coil current. The variables $u^\text {d}_i$, $i=1,2,\ldots ,N^\text {d}$, and $u^\text {c}$ are the input data for the computations. The impedance matrix ${\varvec{Z}}^\text {d}$ is computed in an analogous manner as the algorithm presented for the port treatment on page 3.

### Lower box

We now present a model for $\Psi$, the interior region of the subwoofer’s lower box with dimensions $w\times h_\text {l} \times d$ illustrated in Fig. 2a. The governing equations are Helmholtz equation (1) in $\Psi$ for the acoustic pressure, coupling conditions (4) to the upper box on $\Gamma ^\text {dm}$ and to the exterior on $\Gamma ^\text {p}$, and sound hard (5) boundary condition on $\Gamma ^\text {s}$.

The variational form of the governing equation to compute *p* in $\Psi$ is

32 $$\begin{aligned} \int _{\Psi } \nabla q \cdot \nabla p - k^2\int _{\Psi } qp -\mathrm {i} k\rho c \int _{\Gamma ^\text {dm}} u^\text {d}\cdot n^\text {d} q -\mathrm {i} k \rho c \int _{\Gamma ^\text {p}} u^\text {p}\cdot n^\text {p} q =0 \quad \forall q \in H^1(\Psi ), \end{aligned}$$

Here, we assume that acoustic pressure *p* is planar symmetric in the z-direction. In Fig. 2b, $\psi$ denotes the vertical cross section of $\Psi$, and similarly $\gamma ^\text {p}$ and $\gamma ^\text {d}$ denote the vertical cross section of $\Gamma ^\text {p}$ and $\Gamma ^\text {dm}$ in 2D, respectively. The coupling with the upper box is through the impedance matrix ${\varvec{Z}}^\text {d}$, computed by algorithm presented in the previous section, and the coupling with the output port is through the impedance matrix ${\varvec{Z}}^\text {p}_\text {2D}$.

The output port for the hybrid model is divided into $N^\text {p}_h$ depth running, non overlapping strips $\gamma ^\text {p}_j\times (0,d/2)$ and the acoustic velocity is piece-wise constant on each strip $\gamma ^\text {p}_j$. Then, the impedance matrix ${\varvec{Z}}^\text {p}_\text {2D}$ of order $N^\text {p}_h$ is assembled using the same algorithm as for impedance matrix ${\varvec{Z}}^\text {p}_\text {3D}$. Similarly, the acoustic velocity is piece-wise constant on each strip on the boundary $\gamma ^\text {d}$ and each strip has the area $A_\text {2D}= d/2\left| \gamma ^\text {d}_j\right|$. The corresponding panels on boundary $\Gamma ^\text {dm}$ shown in Fig. 3b has area $A_\text {3D}=\left| \Gamma ^\text {dm}_j\right|$. To obtain the same flow rate from the speaker in 2D, as we have in the 3D model of the upper box, we use the volumetric flow rate equation. We denote the area ratio ($A_\text {3D}/A_\text {2D}$) by $\sigma$ and for each panel *j*, $\sigma _j=2\left| \Gamma ^\text {dm}_j\right| \bigg /\left| \gamma ^\text {d}_j\right| d$.

Under the above assumptions, the variational form for 3D problem in $\Psi$ is reduced to the following 2D problem of finding $p\in H^1(\psi )$ such that

33 $$\begin{aligned} \int _{\psi } \nabla q \cdot \nabla p -k^2 \int _{\psi } qp - \mathrm {i} k \rho c \sum ^{N^\text {d}}_{j=1} u^\text {d}_{j} \sigma _j \int _{\gamma ^\text {d}_j} q -\mathrm {i} k \rho c \sum ^{N^\text {p}_h}_{j=1} u^\text {p}_j \int _{\gamma _j^\text {p}} q = 0, \quad \forall q \in H^1(\psi ). \end{aligned}$$

We triangulate $\psi$ and define *Q* basis functions $\vartheta _1,\,\vartheta _2,\,\ldots ,\,\vartheta _Q$. For the numerical experiments in this paper, we use standard continuous, elementwise bi-quadratic basis functions. Here, we determine ${\varvec{x}}=\big [({\varvec{p}})^T,\,({\varvec{u}}^\text {p})^T,\,({\varvec{u}}^\text {d})^T,\,u^\text {c},\,I \big ]^T$, where ${\varvec{p}}=\big [p_1,\, p_2,\, \ldots ,\, p_Q\big ]^T$, ${\varvec{u}}^\text {p}=\big [u^\text {p}_1,\, u^\text {p}_2,\, \ldots ,\, u^\text {p}_{N^\text {p}_h}\big ]^T$, ${\varvec{u}}^\text {d}$, $u^\text {c}$, and *I* are defined earlier. The discretised form of Eq. (33) is

34 $$\begin{aligned} \sum ^{Q}_{j=1}\left( \int _{\psi }\nabla \vartheta _i \cdot \nabla \vartheta _j- k^2\int _{\psi } \vartheta _i \vartheta _j\right) p_j - \mathrm {i} k \rho c \sum ^{N^\text {d}}_{j=1} u_j^\text {d}\int _{\gamma ^\text {d}_j} \sigma \vartheta _i -\mathrm {i} k \rho c \sum ^{N^\text {p}}_{j=1} u_j^\text {p}\int _{\gamma ^\text {p}_j} \vartheta _i=0, \quad i=1,\,2,\,\ldots ,\,Q. \end{aligned}$$

By putting all the above together, we obtain the following system of governing equations

35 $$\begin{aligned} \begin{bmatrix} {\varvec{A}}^\text {ll} &{} {\varvec{A}}^\text {lp} &{} {\varvec{A}}^\text {ld} &{} {\varvec{0}} &{} {\varvec{0}} \\ {\varvec{A}}^\text {pl} &{} {\varvec{Z}}_\text {2D}^\text {p} &{} {\varvec{0}} &{} {\varvec{0}} &{} {\varvec{0}} \\ {\varvec{A}}^\text {dl} &{} {\varvec{0}} &{} {\varvec{Z}}^\text {dd} &{} {\varvec{Z}}^\text {dc} &{} {\varvec{0}} \\ {\varvec{0}} &{} {\varvec{0}} &{} {\varvec{Z}}^\text {cd} &{} Z^\text {cc} &{} -1 \\ {\varvec{0}} &{} {\varvec{0}} &{} {\varvec{0}} &{} a^\text {Ic} &{} a^\text {II} \end{bmatrix} \begin{bmatrix} {\varvec{p}}\\ {\varvec{u}}^\text {p}\\ {\varvec{u}}^\text {d}\\ u^\text {c}\\ I \end{bmatrix} = \begin{bmatrix} 0\\ 0\\ 0\\ 0\\ V \end{bmatrix}, \end{aligned}$$

where the $Q\times Q$ matrix ${\varvec{A}}^\text {ll}$, the $Q \times N^\text {d}$ matrix ${\varvec{A}}^\text {ld}$, the $Q \times N^\text {p}$ matrix ${\varvec{A}}^\text {lp}$, the $N^\text {p}\times Q$ matrix ${\varvec{A}}^\text {pl}$, and the $N^\text {d}\times Q$ matrix ${\varvec{A}}^\text {dl}$ have entries

36 $$\begin{aligned}&a^\text {ll}_{ij} = \int _{\psi } \nabla \vartheta _i \cdot \nabla \vartheta _j - k^2\int _{\psi } \vartheta _i \vartheta _j, \; a^\text {lp}_{ij} = -\mathrm {i} k \rho c \int _{\gamma _\text {p}^{\left( j\right) }} \vartheta _i, \; a^\text {ld}_{ij} =-\mathrm {i} k \rho c \int _{\gamma _\text {d}^{\left( j\right) }} \sigma \vartheta _i, \; a^\text {pl}_{ij} \nonumber \\&\qquad = \int _{\gamma _\text {p}^{\left( j\right) }}\vartheta _i, \; \text {and}\; a^\text {dl}_{ij} = \frac{d}{2}\int _{\gamma _\text {d}^{\left( j\right) }} \sigma \vartheta _i, \end{aligned}$$

respectively.

## Lumped element model

**Figure 4:**
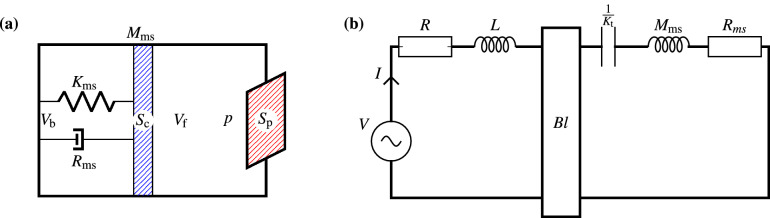
Lumped element model: (**a**) Mass spring damper model of the subwoofer. (**b**) Electromechanical circuit that models the transducer.

We present a fully lumped element model of the subwoofer modelled by the mass-spring-damper system and the electromechanical circuit, illustrated in Fig. 4. We denote the volume of the back and front chamber by $V_\text {b}$ and $V_\text {f}$, respectively. Let $S_\text {c}$ and $S_\text {p}$ be the area, $\delta _\text {c}$ and $\delta _\text {p}$ be the displacement, and $u^\text {c}=\mathrm {i}\omega \delta _\text {c}$ and $u^\text {p}=\mathrm {i}\omega \delta _\text {p}$ be the velocities associated with the speaker cone and the output port, respectively. The mechanical force $F_\text {m}$, the acoustic force $S_\text {c}p$, and the electrical force *BlI* comprise the total force $F_\text {t}=ma=\mathrm {i}\omega M_\text {md}u^\text {c}$ on the speaker cone membrane:

37 $$\begin{aligned} \mathrm {i}\omega M_\text {md}u^\text {c} = F_\text {m} - S_\text {c}p + BlI. \end{aligned}$$

Here, a mass spring damper system models the mechanical force

38 $$\begin{aligned} F_\text {m} = -K_\text {t} \delta _\text {c} - R_\text {ms} u^\text {c} = -K_\text {t} \frac{u^\text {c}}{\mathrm {i}\omega } - R_\text {ms}u^\text {c}, \end{aligned}$$

where $K_\text {t} = C_\text {ms}^{-1}+\rho c^2 S_\text {c}^2 V_\text {b}^{-1}$ is the sum of stiffness due to the speaker and the air in the back chamber.

Now, by substituting the current *I* (9) and the mechanical force $F_\text {m}$ (38) in Eq. (37), we obtain

39 $$\begin{aligned} Z^\text {c} u^\text {c} = - S_\text {c}p + Bl\frac{(V-Blu^\text {c})}{Z^\text {e}}, \end{aligned}$$

where the speaker cone impedance is $Z^\text {c}=\mathrm {i}\omega M_\text {md} + R_\text {ms} + K_\text {t}/{\mathrm {i}\omega }$, and the electrical impedance is $Z^\text {e} = R+\mathrm {i}\omega L$.

We assume an isentropic flow using the ideal gas law, which states that total pressure times volume raised to power $\gamma$ (heat capacity ratio) is constant, which entails that

40 $$\begin{aligned} p = - \rho c^2 \frac{dV_\text {f}}{V_\text {f}} = - \frac{\rho c^2}{V_\text {f}} \left( S_\text {p}\delta _\text {p} - S_\text {c}\delta _\text {c} \right) = \frac{\rho c^2}{\mathrm {i\omega V_\text {f}}}\left( S_\text {c}u^\text {c} - S_\text {p} u^\text {p} \right) , \end{aligned}$$

where *p* is the acoustic pressure, and $dV_\text {f}$ is the change in volume of the front chamber due to movement of speaker cone.

For the lumped model, we assume that $u^\text {p}_1 = u^\text {p}_2 = \cdots = u^\text {p}_{N_\text {p}} = u^\text {p}$ and $p_1 = p_2 = \ldots = p_{N^\text {p}} = p$. Under these assumptions, acoustic impedance relation (10) reduces to

41 $$\begin{aligned} p = Z^\text {p} u^\text {p}, \end{aligned}$$

where $Z^\text {p}=\frac{1}{N^\text {p}}{\varvec{1}}^T{\varvec{Z}}^\text {p}_\text {3D}{\varvec{1}}$ in which $N^\text {p}\times 1$ vector ${\varvec{1}}= [1,\,1,\,\ldots ,\,1]^T$.

From relations (40) and (41), we can write the speaker’s cone velocity as

42 $$\begin{aligned} u^\text {c} = \left( \frac{\mathrm {i}\omega V_\text {f}}{\rho c^2 S_\text {c}} + \frac{S_\text {p}}{S_\text {c}Z^\text {p}} \right) p. \end{aligned}$$

Now, by substituting speaker’s cone velocity $u^\text {c}$ (42) into Eq. (37), we obtain

43 $$\begin{aligned} \Bigg [S_\text {c}+\left( Z^\text {c}+\frac{B^2l^2}{Z^\text {c}}\right) \left( \frac{\mathrm {i}\omega V^\text {f}}{\rho c^2 S_\text {c}}+ \frac{S_\text {p}}{S_\text {c}Z^\text {p}}\right) \Bigg ]p = \frac{BlV}{Z^\text {e}}. \end{aligned}$$

By Eqs. (41), (42), and (43), we can compute the acoustic pressure *p* inside the front chamber, the cone velocity $u^\text {c}$, and the acoustic velocity $u^\text {p}$.

## Validation and discussion

**Figure 5:**
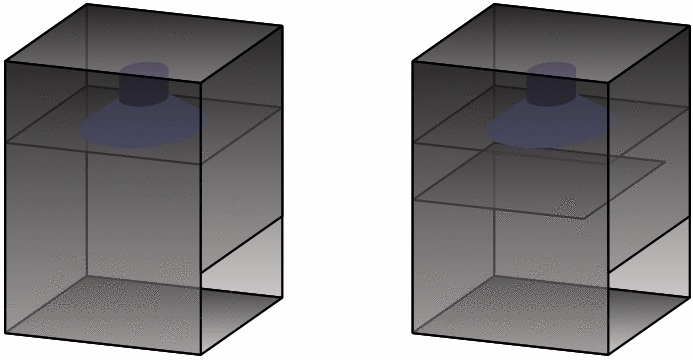
Two subwoofer setups with top-mounted drivers and output ports on the lower right. The subwoofer on the right has an added horizontal wall inside the cabinet.

To validate the results of the hybrid model, we consider two layouts of the subwoofer, illustrated in Fig. 5, with width 80 cm, height 100 cm, and depth 60 cm. The downward-facing 18 in. transducer is mounted on a baffle with dimensions 80 cm $\times$ 60 cm, located 30 cm from the top. The subwoofer layout on the right in Fig. 5 has a 70 cm wide and 2 cm high horizontal wall that is 48 cm from the floor. We use a generic transducer with realistic parameters, as given in Table 1. The input voltage amplitude to the system is $V=1$ V.

The sound pressure level (SPL), 1m in front of the output port, is calculated to assess the performance of the subwoofer. The SPL uses a logarithmic scale to measure the effective pressure of sound relative to a reference pressure in units of decibels (dB). First, we calculate the pressure at 1m by expression

44 $$\begin{aligned} p_\text {1m} = {\varvec{g}}^T{\varvec{u}}^\text {p}, \end{aligned}$$

where $p_\text {1m}$ is the pressure at the floor, 1m in front of the output port, and the $N^\text {p}\times 1$ vector ${\varvec{g}}$ is a vector that is pre-computed by the boundary element method while assembling the corresponding port impedance matrix ${\varvec{Z}}^\text {p}$. Now, we can calculate the SPL of our subwoofer design by

45 $$\begin{aligned} \text {SPL}_\text {1m} = 20\log _{10}\left( \frac{\left| {p}_\text {1m}\right| }{p_\text {o}}\right) , \end{aligned}$$

where $p_\text {o}=20\mu \text {Pa}$ is the standard reference pressure for SPL calculations.

**Table 1:** Parameters of the transducer used for the numerical experiments.

Parameters	Values
Mechanical compliance $\text {C}_\text {ms}$ (mm/N)	0.22
Moving mass $\text {M}_\text {md}$ (g)	150.0
Mechanical resistance $\text {R}_\text {ms}$ (Kg/s)	6.0
Bl factor (N/A)	22.6
Voice coil resistance R $(\Omega )$	5.5
Voice coil inductance L (mH)	1.5

**Figure 6:**
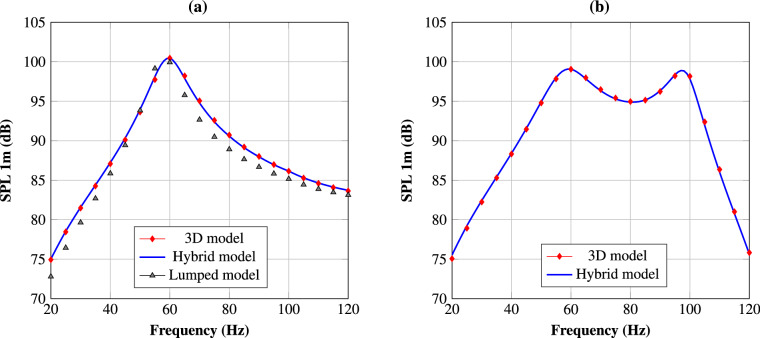
The SPL, 1m in front of the output port, is computed as the function of frequency. (**a**) Results for the subwoofer layout on the left in Fig. 5. (**b**) Results for the subwoofer layout on the right in Fig. 5.

The 3D mathematical model was implemented in COMSOL multi-physics^5^ by using unstructured tetrahedral second order elements with maximum edge length $h = 0.09\,\mathrm {m}$, the hybrid model was implemented in MATLAB^6^ by using uniform square bi-quadratic elements with side length $h=0.01\,\mathrm {m}$, and the lumped model was also implemented in MATLAB. The impedance matrices ${\varvec{Z}}^\text {p}_\text {3D}$ and ${\varvec{Z}}^\text {p}_\text {2D}$ for the output port were computed in PAFEC^7^, and the impedance matrix ${\varvec{Z}}^\text {d}$ for the driver boundary in COMSOL multi-physics. Figure 6 shows the SPL measured as a function of frequency for the subwoofer layouts in Fig. 5. The results of the 3D model closely match the results of the hybrid model for both subwoofer layouts. This validates our assumption of 2D wave propagation within the lower box. For the subwoofer layout, with no material inside the front chamber, the results of the lumped element model are close to the results of the 3D model and follow the same general trend. However, for the second subwoofer layout, with a horizontal wall inside the front chamber, we only compare the results of the 3D and the hybrid model. The lumped model has much more severe limitations compared to the hybrid and the 3D model; lumped elements may work if the elements can be accurately characterised, but doing so may require 2D or 3D calculations in itself for every new subwoofer layout. Therefore, the hybrid and the 3D model are more convenient to use when evaluating a new subwoofer layout.

The 3D model accurately evaluates the performance of general internal subwoofer layouts, and the hybrid model accurately and efficiently evaluates the performance of general planar-symmetric subwoofer layouts. Traditionally, the lumped element model has been used to evaluate the performance of subwoofers because it is simple and easy to implement. However, its elements require a new model for every new subwoofer layout, and is thus not suitable to simulate complex layouts. For the 3D model, we only need to pre-compute the impedance matrix ${\varvec{Z}}^\text {p}_\text {3D}$ to represent the response of the exterior. For the hybrid model, we need to pre-compute the impedance matrices ${\varvec{Z}}^\text {p}_\text {2D}$ to represent the response of the exterior, but we also need to pre-compute the impedance matrix ${\varvec{Z}}^\text {d}$ to represent the response of the upper box. This is a one time cost we pay to compute the impedance matrices in the hybrid model. Then, to evaluate the performance of a given planar-symmetric subwoofer layout, we only need to solve the 2D problem in the lower box. Given that we have calculated the impedance matrices, the hybrid model is significantly faster in computing the wave propagation problem than the full 3D model. For the experiments in this study, we used a system running Windows 10 with Intel Core i7-4790 CPU @ 3.60 GHz processor and 32 GB installed RAM. We performed 20 simulations for time analysis of the 3D and the hybrid model. For each simulation, we used 21 frequencies in the range 20–120 Hz to evaluate the performance of a given subwoofer layout. When using the 3D model, the minimum time for the simulation was 18 min, the maximum time was 20 min 50 s, and the mean time was 19 min 12 s to evaluate the performance at 21 frequencies for a given subwoofer layout compared to the maximum and the minimum time of 42 and 52.5 s, respectively, and the mean time of 44.1 s for the hybrid model. The difference in computational time is significant between the 3D and the hybrid model when we have to evaluate the performance of large sets of subwoofer layouts at multiple frequencies instead of a single frequency. The memory usage for the 3D model is also higher, as it requires 1.25 GB of memory compared to 0.2 GB for the hybrid model. Moreover, we can easily solve finer mesh resolutions for the hybrid model by using elements with side length of $h/2,\,h/4$, or *h*/8. However, for the 3D model, the system mentioned above runs out of memory when using elements with edge length of *h*/4, as degrees of freedom reach approximately 1.3 million. With a significant reduction in computational time, the hybrid model is especially beneficial for use in an optimisation loop or when evaluating different planar-symmetric subwoofer layouts to choose the best performing layout.
